# The effect of arm weight support on upper limb muscle synergies during reaching movements

**DOI:** 10.1186/1743-0003-11-22

**Published:** 2014-03-04

**Authors:** Martina Coscia, Vincent CK Cheung, Peppino Tropea, Alexander Koenig, Vito Monaco, Caoimhe Bennis, Silvestro Micera, Paolo Bonato

**Affiliations:** 1 Translational Neural Engineering Laboratory, Center for Neuroprosthetics and Institute of Bioengineering, School of Engineering, École Polytechnique Fédérale de Lausanne (EPFL), BM 3210 Station 17, Lausanne CH-1015, Switzerland; 2 Istituto di Neuroscienze del Consiglio Nazionale delle Ricerche (CNR), Pisa, Italy; 3 The Biorobotics Institute, Scuola Superiore Sant’Anna, Pisa, Italy; 4 Department of Brain and Cognitive Sciences and McGovern Institute for Brain Research, MIT, Cambridge, MA, USA; 5 Department of Physical Medicine and Rehabilitation, Harvard Medical School, Boston, MA, USA

**Keywords:** Arm weight support, Upper limb rehabilitation, Muscle synergies, Arm reaching movements

## Abstract

**Background:**

Compensating for the effect of gravity by providing arm-weight support (WS) is a technique often utilized in the rehabilitation of patients with neurological conditions such as stroke to facilitate the performance of arm movements during therapy. Although it has been shown that, in healthy subjects as well as in stroke survivors, the use of arm WS during the performance of reaching movements leads to a general reduction, as expected, in the level of activation of upper limb muscles, the effects of different levels of WS on the characteristics of the kinematics of motion and of the activity of upper limb muscles have not been thoroughly investigated before.

**Methods:**

In this study, we systematically assessed the characteristics of the kinematics of motion and of the activity of 14 upper limb muscles in a group of 9 healthy subjects who performed 3-D arm reaching movements while provided with different levels of arm WS. We studied the hand trajectory and the trunk, shoulder, and elbow joint angular displacement trajectories for different levels of arm WS. Besides, we analyzed the amplitude of the surface electromyographic (EMG) data collected from upper limb muscles and investigated patterns of coordination via the analysis of muscle synergies.

**Results:**

The characteristics of the kinematics of motion varied across WS conditions but did not show distinct trends with the level of arm WS. The level of activation of upper limb muscles generally decreased, as expected, with the increase in arm WS. The same eight muscle synergies were identified in all WS conditions. Their level of activation depended on the provided level of arm WS.

**Conclusions:**

The analysis of muscle synergies allowed us to identify a modular organization underlying the generation of arm reaching movements that appears to be invariant to the level of arm WS. The results of this study provide a normative dataset for the assessment of the effects of the level of arm WS on muscle synergies in stroke survivors and other patients who could benefit from upper limb rehabilitation with arm WS.

## Background

Over the last two decades, robotic systems providing adjustable levels of arm-weight support (WS) have been utilized in the rehabilitation of subjects with neurological conditions such as stroke [1-6]. These systems facilitate the performance of upper limb motor training exercises by partially or totally compensating for the effect of gravity, hence decreasing the magnitude of the joint torques that subjects have to generate to move the arm. Besides, the use of robotic systems that provide subjects with arm WS has been shown to lessen the abnormal coupling of shoulder abductors and elbow flexors often observed in stroke survivors who are affected by severe motor impairments [4,7].

Recent studies have provided preliminary results in regard to how arm WS may modify the kinematics of motion and the activity of upper limb muscles during arm reaching movements [8-10]. In both healthy subjects and stroke survivors, WS devices have been shown to facilitate arm movements by reducing the level of muscle activity needed for reaching, particularly for muscles counteracting the effect of gravity [8,9]. These studies have generally compared free and unsupported planar arm reaching movements with arm reaching movements performed with a level of WS that fully compensated for gravity. However, these studies did not provide a detailed analysis of how the kinematics of motion and the activity of muscles may vary across different levels of arm WS. Moreover, none of these studies has directly examined the impact of arm WS on muscle coordination. The analysis of muscle coordination is important for the purpose of understanding the neural mechanisms underlying the control of movement. This is clinically relevant since muscle coordination is often altered in patients with neurological conditions such as stroke [11-16].

Although previous studies provided preliminary results in support of the hypothesis of preservation of the timing of muscle activations with arm WS [8,9], it is unknown if muscle coordination is also preserved in different WS conditions. The activity of muscles during the performance of upper limb movements has been looked upon as consisting of a tonic and a phasic component that account for arm-weight compensation and movement dynamics, respectively [17-19]. One may therefore deduce that the unloading of the arm would reduce only the tonic component of the muscle activity without affecting the phasic component, thus leading to a change in muscle coordination [8,9]. However, the relationship between the tonic and phasic components of the activity of muscles and how their activations are coordinated during movement is not fully understood.

Muscle coordination has been recently studied by many authors using muscle synergies [20-23]. The study of muscle synergies is based on the assumption that muscles are synergistically co-activated via discrete motor modules of neural origin [24] aimed at fulfilling an elementary biomechanical demand. One way to obtain the muscular compositions of muscle synergies is to apply suitable factorization algorithms to the electromyographic (EMG) signals collected during the performance of different motor tasks [25]. It has been observed that the combination of a few muscle synergies can explain a large extent of the variability in spatiotemporal characteristics of muscle patterns of activation recorded during arm reaching movements [18,19]. Such a modular organization has been looked upon as a strategy employed by the central nervous system (CNS) to reduce the complexity of the control of motion [26-31]. Furthermore, patterns of muscle synergies appear to reflect the degree of motor impairment due to conditions that affect the control of motion such as stroke [13,14,32-34].

In earlier studies, muscle coordination patterns were assessed using different analytic methods [35], such as regressions between the activities of pairs of muscles [36,37] as well as wavelet transformation of the EMG signals to enable analyses in the time-frequency domain [38]. These methods are limited to comparing the patterns of activity across muscles. In contrast, muscle synergies provide researchers with quantitative measures of the contribution of each muscle to all elementary biomechanical demands associated with the performance of motor tasks. Hence, the study of muscle synergies facilitates the understanding of complex, high-dimensional muscle activation patterns.

By analyzing lower-limb muscle synergies, Ivanenko et al. [21] found that body WS minimally affects the timing of muscle-synergy activations related to locomotion, but that the muscular compositions of the muscle synergies are modified, especially at high levels of body WS. The aim of our study was to extend the analyses performed by Ivanenko et al. [21] on lower limb muscles to the activity of upper limb muscles. The effects of different levels of arm WS on upper-limb muscle synergies during the performance of reaching movements have never been systematically investigated before. Preliminary observations concerning the effects of arm WS on the performance of reaching movements in healthy individuals [8,9,39] need to be extended by characterizing limb kinematics, muscular activity, and muscle synergies underlying the performance of the motor task with different levels of arm WS. The results of such characterization would provide researchers and clinicians with a normative dataset to assess the performance of arm reaching movements in stroke survivors. We hypothesize that the kinematics of arm movement is preserved in all WS conditions while, as suggested by previous studies [8,9,17,19,21], WS affects both the temporal activation profiles and the muscular compositions of the upper-limb muscle synergies used for arm reaching.

## Materials and methods

### Participants

Nine right-handed healthy adults (age, 27 ± 3 years; weight, 68 ± 10 kg) were recruited in the study. They had no history of upper-limb injury or skin lesions, cardiovascular or respiratory diseases, or difficulty in understanding instructions. All experimental procedures were carried out in the Motion Analysis Laboratory at Spaulding Rehabilitation Hospital after they were reviewed and approved by the hospital’s Ethics Committee. All participants provided informed consent before the experiments, as required by the Declaration of Helsinki and the hospital’s Ethics Committee.

### Experimental setup

**Figure 1:**
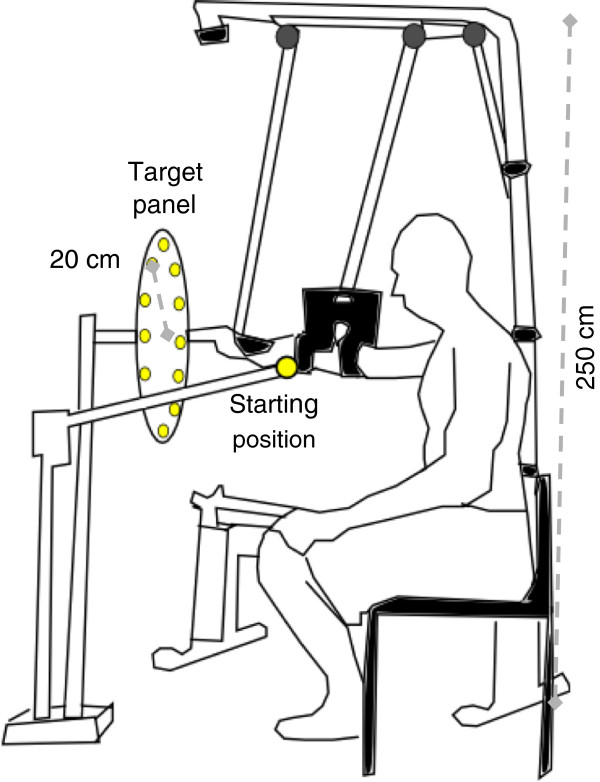
Schematic representation of the experimental setup.

Each subject sat in front of a target panel. The center of the target panel was aligned with the right shoulder acromion (Figure 1). The panel had twelve targets arranged in a clock-like fashion that were positioned 20 cm from its center. The distance between the subject and the center of the panel was set according to each subject’s arm length measured with the fist closed. The hand starting position utilized to perform the arm reaching movements was set along the virtual line connecting the center of the panel and the shoulder acromion and it was located half-way between these points. The Freebal system [2], commercialized as the Armeo Boom by Hocoma AG (Zurich, Switzerland), was utilized to provide subjects with arm WS. The vertical component of the Armeo Boom was aligned with the back of the chair where the subject sat during the experiment and its height was set to 250 cm. The distal end of the horizontal component of the Armeo Boom was aligned with the center of the target panel. Arm WS was provided via two slings for the forearm and upper arm, respectively.

After measuring each subject’s weight and the length of the upper arm and forearm, subjects were instructed to position their arm in the slings of the Armeo Boom device. The level of weight compensation at the upper arm and forearm was adjusted by setting lead-screw sliders. The sliders were used to modify the length of two separate springs that determined the amount of weight compensation provided by each sling. The sliders had nine pre-set positions labeled from A to I that corresponded to the following values of weight compensation: 0.40-0.54 kg for A, 0.67-0.81 kg for B, 0.95-1.09 kg for C, 1.22-1.36 kg for D, 1.50-1.63 kg for E, 1.77-1.91 kg for F, 2.05-2.19 kg for G, 2.32-2.46 kg for H, and exceeding 2.60 kg for I. These values were estimated according to Stienen et al. [40]. We computed the desired level of weight compensation based on the estimated upper-arm and forearm weight (assumed to be 2.3% and 1.5% of the subject’s body weight, respectively [41,42]). The sliders were then set to the position most suitable to match as closely as possible the desired level of WS (40%, 60%, 80% or 100%). In addition to the four levels of arm WS listed above, we also tested subjects with 0% WS. For this condition, the sliders were set to the position labeled as A and the length of the cables connecting the slings to the above-mentioned springs was adjusted to provide the minimum mechanical vertical pull that allowed us to avoid the disengaging of the slings.

The kinematics of motion was acquired at 120 Hz by using an eight-camera motion capture system (Vicon, Oxford Metrics Ltd, Oxford, UK). Twenty spherical reflective markers for motion tracking were placed on specific body landmarks. Four markers were positioned along the body midline on the C7 vertebra, the T10 vertebra, and the superior and inferior ends of the sternum. Eight markers were positioned bilaterally on the anterior superior iliac spine, the shoulder acromion, the lateral epicondyle of the humerus, the midpoint between the shoulder acromion and the lateral epicondyle of the humerus, the radial styloid process, the ulnar styloid process, the midpoint between the lateral epicondyle of the humerus and the ulnar styloid process, and the metacarpophalangeal joint of the middle finger.

Surface EMG signals were recorded from the following 14 muscles of the right arm: triceps brachii (TRI), biceps brachii short head (BICS), biceps brachii long head (BICL), brachialis (BRA), brachioradialis (BRAD), pronator teres (PRO), infraspinatus (INFRA), latissimus dorsi (LAT), upper trapezius (TRAP), rhomboid major (RHO), pectoralis major (PEC), anterior deltoid (DANT), medial deltoid (DMED), and posterior deltoid (DPOS). We followed the Surface Electromyography for Non-Invasive Assessment of Muscles (SENIAM) recommendations [43] for skin preparation and electrode placement.

### Experimental protocol

Before performing the arm reaching trials, a hand-held isometric maximum voluntary contraction (MVC) test was performed for each muscle. This test was performed by the same therapist for all the subjects in order to assure consistency of measurement. During the test of each muscle, subjects were seated and asked to assume a muscle-specific arm posture according to the instructions provided by the therapist. Then, subjects performed a MVC for five times (for the duration of 2 s each time) against the resistance provided by the therapist, taking a 30 s break after each contraction to prevent muscle fatigue.

During the arm reaching trials, subjects were instructed to reach, at a self-selected speed and in a randomized order, the 12 targets on the target panel. The arm reaching movements were performed from the above-described starting position to the selected target and then back to the starting position where subjects stopped and waited for instructions about the next target that they had to reach for. At the starting position, subjects were asked to maintain the whole arm in the transverse plane with the hand and the elbow positioned at shoulder height. Arm reaching trials were performed in six different conditions: free movement without the Armeo Boom (C1) and movements performed using the Armeo Boom with arm WS equal to 0% (C2), 40% (C3), 60% (C4), 80% (C5), and 100% (C6).

### Kinematic analysis

The 3-D trajectory of the reflective marker positioned on the metacarpophalangeal joint of the middle finger of the right hand was used to determine the start and end points of both the center-out (i.e., movement from the starting position to the selected target) and the out-center (i.e., movement from the selected target to the starting position) portions of each trial. The trajectory of this reflective marker was also used for the calculation of hand motion kinematic parameters.

**Figure 2:**
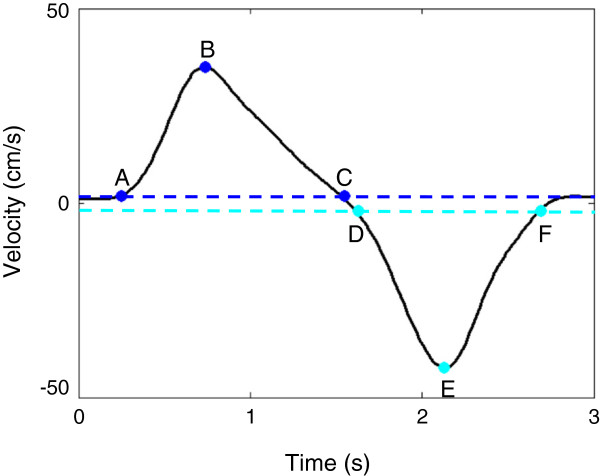
**Hand velocity curve during the performance of an arm reaching trial.** A - Start point, B - maximum velocity, C - end point of the center-out reaching movement. D - Start point, E - minimum velocity, F - end point of the out-center reaching movement. Horizontal dotted lines show how we determined start and end points of each portion (i.e., center-out and out-center) of the arm reaching movement. These lines were set at 5% of the maximum and minimum hand velocity, respectively.

The velocity of movement of the hand was marked by an anti-symmetric bell-shaped curve (Figure 2), with the positive portion of the curve corresponding to the center-out reaching movement, and the negative portion of the curve corresponding to the out-center reaching movement. The start and end points of each center-out reaching movement were defined as corresponding to the times when the hand velocity exceeded, or dropped below, 5% of the maximum value of the velocity profile for that trial (Figure 2, points A and C). Similarly, the start and end points of each out-center movement were defined as the time points at which the velocity profile crossed the threshold line corresponding to 5% of the minimum velocity value for that trial (Figure 2, points D and F).

To compare the quality of movement across testing conditions, the following hand movement kinematic parameters were computed for each trial: (1) accuracy (Ac), defined as the mean value of the minimum distance between each point of the hand trajectory and the straight line connecting the starting position to the target’s position; (2) target distance (TD), defined as the minimum distance between the hand trajectory and the target’s position; (3) maximum peak velocity (MPV), defined as the maximum value of the magnitude of the hand velocity curve; and (4) normalized jerk (NJ), defined as the peak negative jerk value normalized to the maximum of the absolute value of the hand velocity curve [44,45].

Following previous studies [8,9], the kinematics of the upper body was further characterized by using the following angular displacement trajectories: trunk flexion/extension, shoulder flexion/extension, shoulder abduction/adduction, and elbow flexion/extension. The trunk was considered to be at 0 deg when it was perpendicular to the ground. The shoulder was considered to be at 0 deg of both flexion/extension and abduction/adduction when the humerus was parallel to the trunk. Flexion/extension and abduction/adduction were derived by projecting the humerus on the sagittal and coronal planes, respectively. The elbow was considered to be at 0 deg when the arm was completely extended. These anatomical angles were derived from the raw kinematic data using a standard model provided as part of the motion capture system (Vicon, Oxford Metrics Ltd, Oxford, UK).

In order to assess how the level of arm WS affected the above-defined joint angular displacement trajectories, we computed two parameters for each testing condition: the range of motion (ROM_KIN_) and the mean value of each joint angular displacement trajectory (MEAN_KIN_). The MEAN_KIN_ was estimated to control for a potential offset of the joint angular displacement trajectories possibly caused by different levels of arm WS.

### EMG processing

All EMG signals were acquired at 3 kHz, band-pass filtered (40-500 Hz), rectified, low-pass filtered (with a cut-off frequency of 20 Hz), and then integrated over 25-ms intervals to obtain the EMG envelope time series [15]. All filters were implemented as infinite impulse response (IIR) filters based on an elliptic design. For each muscle, the EMG envelope MVC value was defined as the highest value of the EMG envelope time series attained during the MVC test. The EMG data of each muscle recorded for all arm WS conditions was normalized to each muscle’s EMG envelope MVC value to derive normalized EMG envelope time series. These time series were used to estimate the muscle synergies as described below. The normalized EMG envelope time series for each testing condition were segmented into epochs, each containing data of either the center-out or out-center portion of each arm reaching trial. Each epoch was then time-interpolated over 200 points using cubic splines to allow for the comparison of EMG data collected across different WS conditions. In order to estimate changes in muscle activity associated with different levels of arm WS, we estimated the root mean square value of the EMG envelope time series (RMS_EMG_) for each testing condition.

### Muscle synergy estimation

For each subject, the EMG data for the arm reaching movements for all the targets for each WS condition was pooled together in a single matrix and muscle synergies were derived using the non-negative matrix factorization (NNMF) algorithm [46]. Herein, we refer to these muscle synergies identified for each WS condition as condition-specific muscle synergies. The NNMF algorithm models the activity of multiple muscles as a linear combination of several muscle synergies (or vectors, with non-negative components, in the space of the EMG envelope time series), each activated, through multiplication, by a non-negative activation coefficient that varies over time [14,15,32,33,47]. Since the algorithm is formulated to update the solution iteratively starting from an initial random solution until the EMG envelope-reconstruction error reaches a local minimum rather than the global minimum, each synergy extraction was repeated 50 times, and the repetition with the solution explaining the highest overall amount of variance (R^2^) of the EMG envelope time series was selected for further analyses. The number of muscle synergies extracted (i.e., the dimensionality of the muscle-activity subspace identified by the algorithm) was selected to be the minimum number for which an R^2^ ≥ 75% was achieved [14].

For ease of analysis and visualization, each condition-specific muscle synergy extracted from the EMG envelope time series was matched to one in a set of reference muscle synergies that resulted in the highest scalar product between the two vectors [47]. The set of reference muscle synergies was obtained as follows. Since we observed that the number of muscle synergies composing the EMG envelope time series was the same across all WS conditions, we extracted this same number of synergies, using the NNMF algorithm, from a dataset obtained by pooling together the EMG data for all WS conditions. Hence, for each subject we obtained a set of muscle synergies summarizing the features shared across all WS conditions. Then, the synergies obtained in such a way from all subjects were categorized into groups with a hierarchical clustering procedure based on minimizing the Minkowski distance between vectors [15]. The number of clusters specified for this clustering technique was the same as the number of muscle synergies extracted. Finally, the set of reference muscle synergies was obtained by averaging, across subjects, the synergy vectors within each cluster.

The similarity among the condition-specific synergies across WS conditions and the set of reference muscle synergies was assessed using the scalar product (DOT_SYN_). The effect of different levels of arm WS on the temporal activation components of the muscle synergies was assessed using the root mean square (RMS_SYN_) value of the temporal activation components.

### Statistical analyses

The effect of different levels of arm WS on the hand kinematic parameters (Ac, TD, MPV, and NJ), joint angular displacements (ROM_KIN_ and MEAN_KIN_), EMG envelopes (RMS_EMG_), and muscle synergies (DOT_SYN_ and RMS_SYN_) was assessed using repeated measures ANOVA tests (α = 0.05). For the muscle-synergy vectors, statistical analyses using the repeated measures ANOVA test were performed for each muscle component of the muscle synergies. Repeated measures ANOVA tests that showed a statistically significant difference among conditions were followed by post-hoc analyses performed using the Tukey’s honest significant difference test.

## Results

### Changes in upper limb kinematics did not show distinct trends with the level of arm WS

**Figure 3:**
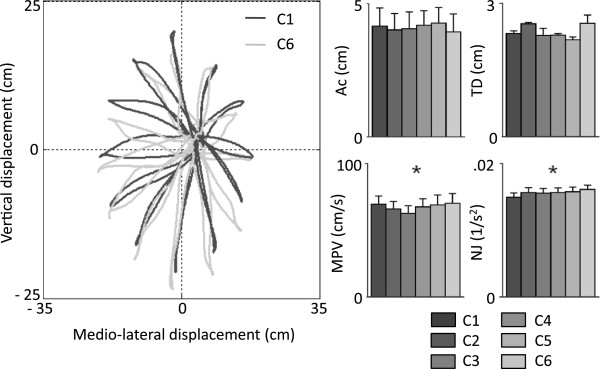
**Hand trajectories and kinematic parameters in different WS conditions.** Hand trajectories in the coronal plane for C1 and C6 (left panel). Kinematic parameters of hand motion (right panel). Ac - accuracy, TD - target distance, MPV - maximum peak velocity, NJ - normalized jerk. For each parameter, each bar height represents the average value across subjects and targets and the error bar represents the standard error. A grey scale is used to show the results for different WS conditions, as reported in the legend.

The characteristics of the hand trajectories for C1 and for the trials when the Armeo Boom was used to provide WS (C2-C6) were found to be very similar in shape. Figure 3 shows the hand trajectory of motion projected onto the coronal plane for C1 (when arm reaching movements were performed without using the Armeo Boom) and for C6 (when arm reaching movements were performed using the Armeo Boom with settings producing 100% WS). Visual inspection of the hand trajectories of motion for all the testing conditions showed no major differences across levels of arm WS. This observation was confirmed via statistical analysis of hand trajectory kinematic parameters. The accuracy (Ac) and target distance (TD) values showed no statistically significant differences across levels of arm WS. The maximum peak velocity (MPV) and the normalized jerk (NJ) parameters showed significant differences across testing conditions, but the magnitude of such differences was modest. Post-hoc analyses revealed statistically significant differences (of modest magnitude) in MPV values for C3 vs. C6 as well as in NJ values for C1 vs. C6.

**Figure 4:**
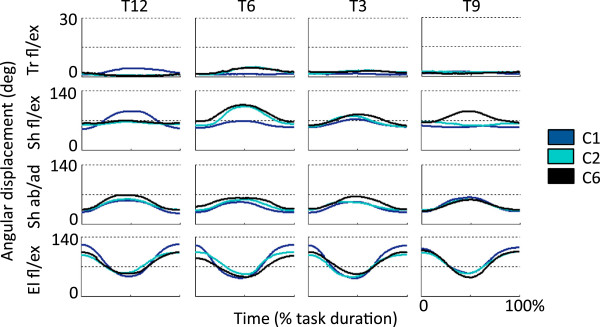
**Angular displacement trajectories for the trunk flexion/extension (Tr fl/ex), the shoulder flexion/extension (Sh fl/ex), the shoulder abduction/adduction (Sh ab/ad), and the elbow flexion/extension (El fl/ex) in three different arm WS conditions (i.e., C1, C2, and C6).** Data is displayed for arm reaching movements to four targets: the north target, T12; the south target, T6; the east target, T3; and the west target, T9. For each plot, the tick lines represent the average (across subjects) angular displacement trajectories.

Visual inspection of the angular displacement trajectories for the trunk, shoulder and elbow (Figure 4) suggested that the level of arm WS affects the kinematics of motion. However, no distinct trend was observed in association with the level of arm WS. Trunk movements were very limited in magnitude, spanning a range that was generally smaller than 5 deg. Shoulder flexion/extension and shoulder abduction/adduction trajectories spanned a range of approximately 30 deg. Patterns of motion were more repeatable for abduction/adduction movements compared to flexion/extension movements. Elbow flexion/extension movements were the ones of larger magnitude compared to all joint movements considered in the study. Elbow flexion/extension movements generally spanned a range of approximately 60 deg.

**Figure 5:**
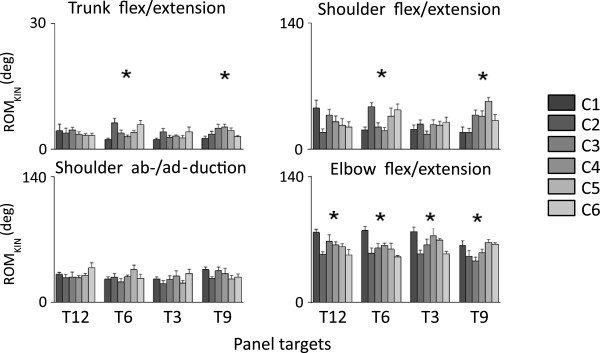
**ROM**_**KIN **_**for the trunk flexion/extension, shoulder flexion/extension, shoulder abduction/adduction, and elbow flexion/extension.** Data is shown for arm reaching movements to four targets: the north target, T12; the south target, T6; the east target, T3; and the west target, T9. A grey scale is used to show the results for different WS conditions, as reported in the legend.

These qualitative observations were confirmed by quantitative analyses performed on the ROM_KIN_ and the MEAN_KIN_ values obtained for different WS levels of each of the considered joint angular displacement trajectory. ROM_KIN_ data (Figure 5) was marked by variations across WS conditions, but such variations did not appear to correlate with the level of WS. Repeated measures ANOVA tests showed statistically significant differences among WS conditions for the trunk, the shoulder flexion/extension, and the elbow flexion/extension ROM_KIN_ data. Differences in trunk flexion/extension ROM_KIN_ data across levels of arm WS were limited to 2-3 deg and hence they were not considered to be biomechanically relevant. Larger differences (i.e., 15-20 deg) were observed for the shoulder flexion/extension ROM_KIN_ data. These differences were found to be statistically significant for target 6 and target 9. However, no distinct association with the level of arm WS was apparent. Statistically significant differences (of 20-25 deg) were observed for the elbow flexion/extension ROM_KIN_ data across levels of arm WS for all the targets. Post-hoc tests showed that the elbow flexion/extension ROM_KIN_ data for C1 tended to be greater in magnitude than the elbow flexion/extension ROM_KIN_ data for other WS conditions. However, no other distinct trends were observed across levels of arm WS. Similar conclusions were drawn from the MEAN_KIN_ data (not shown). Marginal changes across levels of arm WS were observed for the trunk flexion/extension MEAN_KIN_ data. No statistically significant differences were observed for the shoulder abduction/adduction and the elbow flexion/extension MEAN_KIN_ data. Statistically significant differences were observed for the shoulder flexion/extension MEAN_KIN_ data for target 9 with a general trend toward an increase in shoulder flexion with the increase in the level of arm WS. However, this trend was not observed for other targets.

### The level of arm WS affected the amplitude of the EMG data

**Figure 6:**
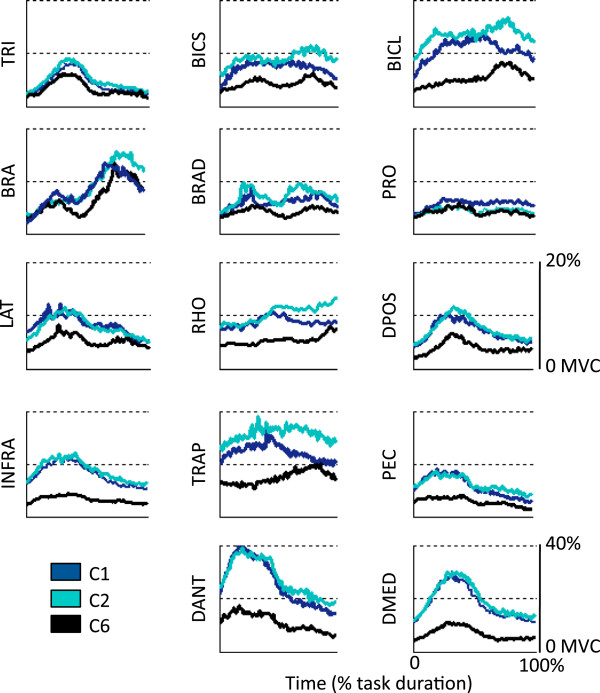
**Normalized EMG envelope time series averaged across subjects and targets for all muscles monitored during the study.** The data is shown for three testing conditions (C1, C2 and C6) and for the following muscles: triceps brachii – TRI; biceps brachii short head – BICS; biceps brachii long head – BICL; brachialis – BRA; brachioradialis – BRAD; pronator teres – PRO; latissimus dorsi – LAT; rhomboid major – RHO; posterior deltoid – DPOS; infraspinatus – INFRA; upper trapezius – TRAP; pectoralis major – PEC; anterior deltoid – DANT; and medial deltoid - DMED.

**Figure 7:**
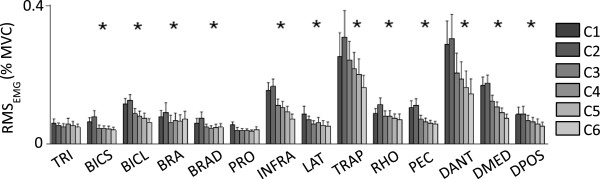
**RMS**_**EMG **_**values for all muscles monitored during the study and for all testing conditions (C1 to C6).** The data is shown for the following muscles: triceps brachii – TRI; biceps brachii short head – BICS; biceps brachii long head – BICL; brachialis – BRA; brachioradialis – BRAD; pronator teres – PRO; infraspinatus – INFRA; latissimus dorsi – LAT; upper trapezius – TRAP; rhomboid major – RHO; pectoralis major – PEC; anterior deltoid – DANT; medial deltoid – DMED; and posterior deltoid – DPOS.

Figure 6 shows average (across subjects and targets) normalized EMG envelope time series for all muscles monitored during the arm reaching trials. The plots show the EMG data for the center-out and the out-center portions of the arm reaching movement as a single curve. Visual inspection of the EMG data revealed that four muscles (i.e., the anterior deltoid - DANT, the medial deltoid - DMED, the infraspinatus - INFRA, and the upper trapezius - TRAP) were recruited at a higher level of their MVC compared to the remaining muscles that were monitored during the arm reaching trials. This pattern of activation across the monitored muscles was also apparent from the RMS_EMG_ values estimated across WS conditions (Figure 7).

Several muscles (i.e., triceps brachii - TRI, latissimus dorsi - LAT, infraspinatus - INFRA, and the compartments of the deltoid - DANT, DMED, and DPOS) showed a peak of activity during the center-out portion of the arm reaching movement. The pectoralis major (PEC) was also predominantly active during the center-out portion of the arm reaching movement. The brachialis - BRA and brachioradialis – BRAD muscles showed two peaks of activity roughly corresponding to the midpoint of the center-out and the out-center portions of the arm reaching movement, respectively. Few muscles were predominantly active during the out-center portion of the arm reaching movement (i.e., biceps brachii long head - BICL and rhomboid major - RHO). The remaining muscles (biceps brachii short head - BICS, pronator teres - PRO, and upper trapezius – TRAP) were generally active throughout the whole arm reaching movement.

Figures 6 and 7 also show that an increase in arm WS was associated, as anticipated, with a reduction in the amplitude of the normalized EMG envelope time series for several muscles. Such amplitude reduction was prominent for the biceps brachii long head - BICL, infraspinatus - INFRA, upper trapezius - TRAP, pectoralis major - PEC, and the compartments of the deltoid - DANT, DMED, and DPOS. The timing of activation of the majority of the monitored muscles appeared to be preserved across WS conditions. We note that using the Armeo Boom device (i.e., C1 vs. C2) only minimally affected the amplitude of most of the EMG envelope time series. For the rhomboid major - RHO, upper trapezius - TRAP, and biceps long and short heads - BICL and BICS the levels of activation during the out-center portion of the arm reaching movement appeared to be slightly higher for C2 compared to C1.

Repeated measures ANOVA tests performed on the RMS_EMG_ values for each muscle across WS conditions confirmed the significant effect of arm WS on the level of activity of all muscles with the exception of the triceps brachii – TRI, and the pronator teres - PRO. Post-hoc analyses showed that RMS_EMG_ values for C1 and C2 were generally higher than RMS_EMG_ values for other levels of arm WS.

### Arm WS did not change the muscle synergies but reduced the amplitude of their temporal activations

**Figure 8:**
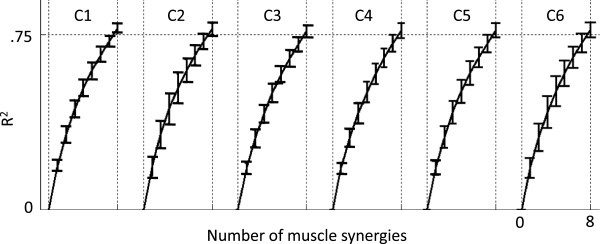
**R**^**2**^**vs. number of muscle synergies for each testing condition (C1 to C6).**

Eight muscle synergies (S1 to S8) were extracted for all subjects and WS conditions. The number of synergies was chosen so that they accounted for at least 75% of the variance (i.e., R^2^ ≥ 75%) of the EMG envelope time series for different WS conditions. The number of synergies chosen using this criterion was consistent across subjects and WS conditions (Figure 8).

**Figure 9:**
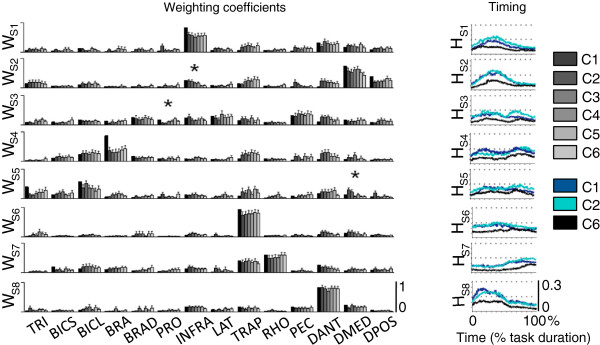
**Weighting coefficients (W**_**S1**_**to W**_**S8**_**) and temporal activations (H**_**S1**_**to H**_**S8**_**) for all eight muscle synergies identified during the study.** The black bars representing weighting coefficients show the set of reference muscle synergies derived by pooling together the EMG data for all WS conditions (see text for details). Weighting coefficients are shown for each arm WS conditions (C1 to C6). Temporal activations are shown for C1, C2 and C6; they are averaged across subjects and targets. The data is shown for the following muscles: triceps brachii – TRI; biceps brachii short head – BICS; biceps brachii long head – BICL; brachialis – BRA; brachioradialis – BRAD; pronator teres – PRO; infraspinatus – INFRA; latissimus dorsi – LAT; upper trapezius – TRAP; rhomboid major – RHO; pectoralis major – PEC; anterior deltoid – DANT; medial deltoid – DMED; and posterior deltoid – DPOS.

The muscular compositions of the extracted muscle synergies were preserved across different levels of WS: the same eight muscle synergies were identified for all WS conditions (Figure 9). The synergies from each condition matched well the set of reference muscle synergies (Figure 9, black bars) extracted from the data matrix containing all EMG envelope data from all WS conditions.

The extracted muscle synergies showed the following characteristics:

• S1 mainly involved the infraspinatus - INFRA, the anterior and medial deltoid - DANT and DMED and the upper trapezius - TRAP. It was primarily recruited during the center-out portion of the arm reaching movement. This synergy contributed to performing forward elevation of the arm while elevating the shoulder and abducting and externally rotating the upper arm.

• S2 mainly involved the medial deltoid - DMED and the upper trapezius - TRAP. It also involved, but to a smaller degree, the anterior deltoid (DANT), the posterior deltoid (DPOS), the infraspinatus – INFRA and the triceps brachii – TRI. S2 was mainly recruited during the center-out portion of the arm reaching movement. This synergy facilitated forward elevation of the arm and extension of the elbow.

• S3 mainly involved the pectoralis major - PEC, latissimus dorsi - LAT, infraspinatus - INFRA, upper trapezius - TRAP and brachioradialis - BRAD. Its activation was marked by two peaks of activity occurring at the end of the center-out portion of the arm reaching movement and at the beginning of the out-center portion of the movement. This synergy facilitated achieving stabilization and postural support of the arm.

• S4 mainly involved the biceps brachii long head - BICL and the brachialis - BRA. This synergy also involved, though to a lesser extent, the upper trapezius - TRAP and the anterior deltoid – DANT. Its activation showed a peak during the center-out portion of the arm reaching movement and one during the out-center portion of the movement. It facilitated flexing the elbow while maintaining the shoulder in flexion.

• S5 mainly involved the biceps brachii long head - BICL and the triceps brachii – TRI. The biceps brachii short head - BICS, upper trapezius - TRAP, and anterior deltoid (DANT) also contributed to this synergy, but to a lesser extent. The timing of activation of this synergy was similar to that of S3 and S4. This synergy appeared to facilitate achieving stabilization of the arm.

• S6 was a muscle-specific synergy dominated by the activity of the upper trapezius – TRAP, with minor contributions from other muscles.

• S7 mainly involved the upper trapezius - TRAP and rhomboid major - RHO. It was activated during the out-center portion of the arm reaching movement. This synergy facilitated stabilizing the shoulder while controlling the position of the hand during the final part of the out-center portion of the arm reaching movement.

• S8 was a muscle-specific synergy dominated by the activity of the anterior deltoid – DANT with minor contributions from other muscles.

The level of arm WS did not generally affect the weighting coefficients of the muscle synergies. Only in few cases (Figure 9), repeated measures ANOVA tests revealed a statistically significant difference across arm WS conditions. In those few cases, we observed that the muscles for which statistical significant differences across WS conditions were observed contributed very modestly to the corresponding muscle synergy. Hence, the biomechanical contributions of these muscles within their corresponding muscle synergies were considered to be negligible [20].

The effect of the level of arm WS on the temporal activation patterns of the muscle synergies (Figure 9) was similar to the effect observed on the EMG envelope time series. An increase in arm WS led to a reduction in the level of activity of all muscle synergies. The shape of the temporal activation patterns was generally preserved with arm WS.

**Figure 10:**
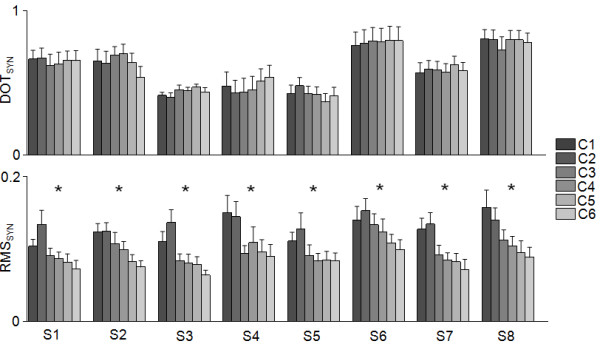
**The effect of different levels of arm WS on muscle synergies.** Top panel - Scalar products (DOT_SYN_) between the weighting coefficients of the reference muscle synergies and the weighting coefficients of the muscle synergies for all WS conditions (C1 to C6). Bottom panel - RMS_SYN_ of the temporal activations (H_S1_ to H_S8_ in Figure 9) for all WS conditions (C1 to C6).

Statistical analysis of the weighting coefficients and the temporal activations of the muscle synergies for different levels of WS confirmed the above-summarized observations derived from visual inspection of the muscle synergy data. The similarity between the synergy set derived for each WS condition and that derived for the set of reference muscle synergies (Figure 9, black bars) was high across all testing conditions (see DOT_SYN_, Figure 10). In addition, repeated measures ANOVA tests showed no statistically significant differences in the DOT_SYN_ values for different levels of arm WS. The level of activity of the temporal activations was significantly affected by the level of arm WS and generally decreased with increasing levels of WS (see RMS_SYN_, Figure 10). Repeated measures ANOVA tests showed statistically significant differences across levels of arm WS for all eight muscle synergies. Post-hoc analyses generally showed that the levels of activity of the temporal activations for C1 and C2 were greater than for the other testing conditions.

## Discussion

### Changes in upper limb kinematics do not show distinct trends with the level of arm WS

The analysis of the hand trajectory and the joint angular displacement trajectories for the trunk, shoulder and elbow showed no distinct trends with the level of arm WS provided during the performance of arm reaching movements. This observation is in general agreement with previous studies that reported no modification in hand trajectory [48,49], area of the upper-limb workspace [4] and symmetry [9] in arm reaching movements performed by healthy subjects under different levels of gravity compensation. However, it is worth noting that some discrepancies exist among the results reported in previous studies. Papaxanthis et al. [48] reported that movement duration and peak velocity did not change when compensating for the effect of gravity. Prange et al. [9] also reported no significant changes in reaching time with gravity compensation. However, Nielsen et al. [49] observed a significant increase in movement duration and a decrease in hand peak velocity during arm reaching movements performed in reduced gravity conditions. Furthermore, Nielsen et al. [49] observed that reducing the effect of gravity altered the kinematics of movement with participant-specific changes.

The slight discrepancies among previous studies and between previously reported results and the results of our own study might be, at least in part, attributed to the different techniques utilized by different authors to compensate for the effect of gravity on upper limb movements. Papaxanthis et al. [48] assessed the effect of gravity by asking subjects to move the limb in the direction of gravity (i.e., downward) and then in the direction against gravity (i.e., upward). Nielsen et al. [49] minimized the effect of gravity by changing body orientation. Prange et al. [9] used the same device that we utilized in our study to provide arm WS. The results reported by Prange et al. [9] and our results appear to be consistent in suggesting that the Armeo Boom allows one to compensate for the effect of gravity without systematically affecting the kinematics of movement. It is worth emphasizing that differences were observed in the kinematics of movement among levels of arm WS. However, such differences did not show a distinct trend with the level of arm WS. A possible interpretation of these results is that healthy subjects may be able to adapt to the level of arm WS provided by the device in a variety of different ways, namely by using different biomechanical strategies. Hence, the variability in the kinematics of movement that we observed in our study for a given level of arm WS across individuals and the lack of a distinct relationship between the kinematics of upper limb movement and the levels of arm WS.

It is important to emphasize that, although we did not observe any systematic change in the kinematics of arm motion with the level of arm WS in healthy subjects, we would anticipate observing a different behavior in patients with neurological conditions that affect the performance of upper limb movements such as stroke. In fact, upper limb control in these patients is often affected by an abnormal coupling of shoulder abductors and elbow flexors [4,7]. The arm WS is expected to have a significant beneficial effect on such abnormal coupling thus improving the performance of arm reaching movements [7,10,50-52]. Besides, the arm WS is expected to have a significant beneficial impact on the smoothness of arm movements, possibly due to an improvement in shoulder-elbow coordination [48,49,53]. These observations emphasize that different motor behaviors must be expected in stroke survivors compared to the ones observed in this study in healthy subjects. In fact, the results of this study are not meant to be generalized to stroke survivors, but rather they are meant to serve as a reference data set to assess responses to different levels of arm WS in stroke survivors.

### A general relationship between muscle-activity amplitude and level of arm WS

Not surprisingly, the level of arm WS had a significant effect on the EMG data collected from the antigravity muscles responsible for the elevation of the shoulder, and the abduction and ante-flexion of the arm. Our observations suggested the preservation of the timing of activation of all upper limb muscles monitored during the experiments across arm WS levels [8,9], with a progressive reduction in amplitude of EMG activity with the level of arm WS. In this study, we used five different levels of arm WS (C2 to C6) and tested subjects also during the performance of free (i.e., no attachment to the slings) arm reaching movements (C1). Previous studies were generally limited to testing fewer arm WS conditions. In our study, arm reaching movements were performed in a 3-D space whereas in previous studies movements were generally performed in a 2-D plane. Besides, in our study, we recorded the activity of a sizable number of muscles. A smaller number of muscles was generally considered in previous studies. Hence, we see our results as strong evidence of the relationship between the amplitude of muscle activity and the level of arm WS.

### Muscle synergies: robustness and possible biomechanical functions

Our factorization analysis showed that the combination of eight muscle synergies explained a large extent of the variability of muscle patterns recorded during reaching to different directions, under all WS conditions. Contrary to our initial hypothesis, the compositions of the muscle synergies were robust to changes in arm WS. Such robustness of the modular structure of the muscle patterns supports the hypothesis that the muscle synergies found in our analysis represent the basic modules employed by the motor system to generate arm reaching movements.

The invariance in the compositions of the muscle synergies might appear to be in conflict with the variability in the angular displacement trajectories observed across levels of arm WS. However, it must be emphasized that the above-reported analyses of the angular displacement trajectories were carried out separately for movements performed to reach for each of the targets on the target panel. In contrast, muscle synergies were derived by processing aggregate EMG data collected when subjects reached for all the targets. Hence, muscle synergies - as we derived them in our study - captured motor strategies that are invariant across movements performed to reach for different targets.

The eight muscle synergies that we identified in the study could be further categorized into postural and movement-related muscle synergies [17-19]. This distinction is possible because joint torques for arm movements can be decomposed into a component that scales in proportion to the movement speed, and another that counteracts gravity [54,55]. In fact, Flanders and colleagues found that muscle activity during planar reaching movements towards three different directions could be decomposed into two components: a tonic component aimed at maintaining postural stability and a phasic component aimed at controlling movement [17]. Also, d’Avella and colleagues, who investigated muscle synergies underlying arm reaching movements performed at different speeds [19], found that three muscle synergies capture the activity of postural muscles and that such tonic synergies are invariant to the speed of the arm reaching movements. Future studies (including the performance of arm reaching movements at different speeds) could further investigate the eight muscle synergies herein reported by relating their activations to their tonic and phasic functions and by assessing their movement speed dependence.

Our results showed a relatively low degree of dimensionality reduction provided by the muscle synergies. In our study, the variability in 14 EMG envelope time series was explained by 8 muscle synergies. Previous studies that analyzed muscle synergies for arm reaching movements showed a higher degree of dimensionality reduction. Flanders and colleagues found 2 muscle synergies from 9 muscles [17]. Sabatini found 2 or 3 synergies from 6 muscles [20]. d’Avella and colleagues found 5 or 6 synergies from 19 muscles [18,19]. Cheung and colleagues found 7 synergies from 16 muscles [15]. The above-referenced results are not directly comparable with ours and among them because of the differences in the muscles monitored in each study, the task contingencies, the EMG pre-processing procedures, and the factorization method utilized by different authors in different studies. Nonetheless, we noticed that one factor contributing to the low degree of dimensionality reduction is the presence of muscle-specific synergies, or synergy vectors dominated by single muscles (i.e., synergies S6 and S8). Muscle-specific synergy vectors have been previously reported [20]. How these muscle-specific synergy vectors should be interpreted is a topic under discussion. Such synergies could represent muscle-specific control commands generated by the CNS [56]. However, some authors consider them to be artifacts arising from the assumptions behind the specific factorization algorithm used. In fact, Sabatini excluded any muscle-specific synergy from the analysis of his results [20].

### A central representation of the gravitational force?

Our analysis of the temporal activations of the muscle synergies shows that for all synergies, the amplitude of activation decreased as the level of arm WS increased. Specifically, the analysis of the RMS_SYN_ highlights that the level of activation of the muscle synergies was generally similar for C1 and C2, but decreased with increasing levels of arm WS. This reduction in the level of activation of the muscle synergies could reflect a specific control strategy used by subjects in response to the level of arm WS. The CNS may regulate the level of activation of the muscle synergies according to the perceived arm weight (when offset via arm WS) by scaling the amplitude of all the control signals (i.e., the temporal activation components of the muscle synergies). It was, in fact, hypothesized that the CNS controls the upper limb with internal models that incorporate gravity as a parameter to modulate the overall level of muscle activity [57,58]. In this context, our findings suggest a central representation of the gravitational force that influences the motor commands associated with the execution of upper limb movements.

The scaling in the amplitude of muscle synergy activation profiles associated with different levels of arm WS is analogous to the scaling in amplitude of the activity of muscles that generate isometric forces of different magnitudes for a given motor task. Previous studies on fingertip force generation [59,60] and isometric force generation by the hand [61] showed that different force magnitudes are generated by linear scaling of the activations of muscular coordination patterns. In fact, the similarity in synergy structure and timing of synergy activation between our study and the study by Roh et al. [61] suggests that motor output changes in response to different levels of arm WS and the generation of isometric forces by the hand may be based on the same underlying control mechanisms.

### Muscle synergy as a possible marker for identifying an optimal level of arm WS for rehabilitation

Robotic systems providing WS for upper limb rehabilitation are able to positively affect the abnormal coupling between the shoulder and elbow thus increasing the working area of the hemiparetic arm [7,10,50-52]. Interestingly, Ellis et al. [7,62] found that providing partial arm WS led to larger improvements in stroke survivors than providing support to the entire limb weight. However, in these studies the evaluation of the performance of arm reaching movements was exclusively based on the kinematics of movement without including any measures of neural signals, thus precluding researchers from achieving a thorough understanding of the mechanisms underlying the observed functional improvements.

A recent study investigating changes in the biomechanics of movement and the muscular activations induced by an arm WS device in stroke survivors reported a reduction in activity in the muscles needed for reaching, particularly in those muscles that counteract the effect of gravity [8]. The authors analyzed the activity of few muscle groups recorded while subjects executed planar reaching movements with total or no arm support. The authors did not investigate the effect of partial levels of arm WS on muscle coordination. Since providing arm WS positively affects the rate of recovery of upper limb control [7,62], one could speculate that the motor recovery process could be associated with changes in the muscle coordination patterns that underlie the control of upper limb joints.

The results herein presented suggest that the analysis of muscle synergies provides researchers with a viable framework to study muscle coordination changes in response to different levels of arm WS. The analyses herein presented could be extended to characterize how stroke survivors may or may not change their muscle coordination as a function of the level of arm WS. Given that rehabilitation with partial WS may induce greater functional improvements than one with full WS [7,62], it is not unreasonable to hypothesize that stroke survivors may deploy a different set of muscle synergies only when the level of arm WS is set to a specific level. This level of arm WS may well indicate an optimal level of weight compensation that would confer the best therapeutic effect. The set of muscle synergies identified from the EMG data may serve as a physiological marker [14] for identifying the optimal setting for a rehabilitative intervention.

### Study limitations

In our study, subjects performed reaching movements at a self-selected speed. We assumed that the modifications in EMG activity were to be attributed only to the different levels of arm WS, but we acknowledge that movement speed may influence the amplitude of EMG activity and should be controlled in future experiments involving a WS device.

Other authors [18,19] have also pointed out that by extracting time-invariant muscle synergies from the EMG signals, the resulting temporal activation of each muscle synergy would potentially include both the phasic and tonic components of the muscle activity. We acknowledge that tonic muscle activities may contribute to the activations of several muscle synergies. However, to explicitly incorporate the tonic and phasic components into our model of motor-output generation would require the formulation of a new algorithm. This could be a potentially fruitful line of future research.

## Conclusions

Our experiments and data analyses showed that the kinematics of the upper limb in healthy subjects performing arm reaching movements changed with the levels of arm WS. However, the observed changes were not marked by distinct trends with the level of arm WS. We interpreted this result as an indication that healthy subjects are capable of generating multiple biomechanical strategies to adapt to the level of arm WS. We also observed that the level of arm WS did not alter the composition of the muscle synergies employed by healthy subjects to perform arm reaching movements. However, the amplitude of the activation profiles of muscle synergies decreased as the level of WS increased. These results appear to be consistent with previous observations that supported the hypothesis of an internal model of the effect of gravity utilized by the CNS to generate appropriate patterns of muscle activations. Overall, our results argue for the usefulness of the muscle synergy model as a framework for understanding the effect of different levels of arm WS on muscle coordination during the performance of upper limb therapeutic exercises. Such an understanding is expected to facilitate the most judicious use of arm WS during rehabilitation for the purpose of promoting functional recovery.

## Abbreviations

Ac: Accuracy; BICL: Biceps brachii long head; BICS: Biceps brachii short head; BRA: Brachialis; BRAD: Brachioradialis; CNS: Central nervous system; C1: Testing condition without the Armeo Boom; C2 to C6: Testing conditions with the Armeo Boom and WS equal to 0%, 40%, 60%, 80% and 100%; DANT: Anterior deltoid; DMED: Medial deltoid; DOTSYN: Scalar product between pairs of condition-specific muscle synergies; DPOS: Posterior deltoid; EMG: Electromyographic; HS1 to HS8: Temporal activations of the muscle synergies S1 to S8; IIR: Infinite impulse response; INFRA: Infraspinatus; LAT: Latissimus dorsi; MEANKIN: Mean value joint angular displacement trajectory; MPV: Maximum peak velocity; MVC: Maximum voluntary contraction; NJ: Normalized jerk; NNMF: Non-negative matrix factorization; PEC: Pectoralis major; PRO: Pronator teres; RHO: Rhomboid major; RMSEMG: Root mean square value of the EMG envelope time series; RMSSYN: Root mean square value of the temporal activations of the muscle synergies; ROMKIN: Range of motion; R2: Variance of the EMG envelope time series accounted for by a specific muscle synergy estimate; SENIAM: Surface Electromyography for Non-Invasive Assessment of Muscles; S1 to S8: Muscle synergies; TD: Target distance; TRAP: Upper trapezius; TRI: Triceps brachii; WS: Weight support; WS1 to WS8: Muscular compositions of the muscle synergies S1 to S8.

## Competing interests

The authors declare that they have no competing interests.

## Authors’ contributions

MC carried out experiments, analyzed data and wrote the paper. VCKC analyzed data and wrote the paper. PT designed the study. AK analyzed data and wrote the paper. VM wrote the paper. CB carried out experiments. SM designed the study, analyzed data, and wrote the paper. PB designed the study, analyzed data, and wrote the paper. All authors read and approved the final manuscript.
